# Delivering bad news in clinical practice: the role of communication skills and emotional intelligence among Polish healthcare professionals

**DOI:** 10.1186/s12909-025-08222-3

**Published:** 2025-11-29

**Authors:** Justyna Kosydar-Bochenek, Jacek Kobak, Mateusz Szczupak, Jakub Hubert Będkowski, Sabina Krupa-Nurcek

**Affiliations:** 1 Faculty of Health Sciences and Psychology, Collegium Medicum, University of Rzeszów, Rzeszów, Poland; 2 Department of Otorynolaryngology, Faculty of Medicine, Medical University of Gdańsk, Gdańsk, Poland; 3 Department of Anesthesiology and Intensive Care, Copernicus Hospital, Gdańsk, Poland; 4 Department of Medical Education, Jagiellonian University Medical College, Kraków, Poland; 5 Faculty of Medical Sciences, University of Rzeszów, Rzeszów, Poland; 6 University Center for Research and Development in Health Sciences, University of Rzeszów, Rzeszów, Poland

**Keywords:** Breaking bad news, Communication skills, Emotional intelligence, Healthcare professionals, Patient communication, Poland

## Abstract

Effective communication and emotional intelligence (EI) are essential to ethically grounded, patient-centred care, particularly when delivering bad news (BBN). Despite growing attention in international healthcare education, empirical data from Central and Eastern Europe remain scarce.

**Methods** this cross-sectional study surveyed 929 Polish healthcare professionals (physicians, nurses, paramedics, midwives, and physiotherapists). Standardised instruments included the Health Professionals Communication Skills Scale (HP-CSS), the Emotional Intelligence Questionnaire (INTE), and the Breaking Bad News Attitudes Scale (BBNAS). Data were analysed using descriptive statistics, ANOVA, Pearson’s correlations, and multiple regression.

**Results** nurses, and to a lesser extent physicians, demonstrated stronger communication competencies and higher openness to breaking bad news (BBNAS Openness). Both positive (informative communication, INTE I – using emotions in reasoning) and negative predictors (Respect, INTE II – recognising emotions) were identified. Effect sizes were small (η² ≤ 0.04; adjusted R² = 0.02–0.06), indicating that, although statistically significant, the findings had a limited practical impact.

**Conclusions** the findings underscore the significance of emotional intelligence and practical communication skills in equipping healthcare professionals to manage emotionally demanding interactions effectively. Tailored training integrated into medical education may foster more confident, ethical, and patient-centred BBN practices. However, the modest effect sizes suggest that multiple psychological and organisational factors contribute to readiness.

## Introduction

 Effective communication is at the heart of good medical care. It is a crucial professional competency, grounded in empathy, clarity, and presence. In today’s healthcare environments, where decisions are often made under pressure and emotions run high, the ability to communicate clearly and compassionately is indispensable. Failures in communication remain a leading cause of adverse events and medical malpractice claims [1, 2]. This has led to increasing calls for structured, evidence-based communication training in all healthcare professions.

Few situations test a clinician’s communication skills as profoundly as breaking bad news (BBN). Delivering a terminal diagnosis, informing families of sudden death, or explaining a poor prognosis all sit at the intersection of medicine and humanity. They demand more than medical precision; they require emotional regulation, sensitivity, and moral clarity [3–5]. The ability to manage one’s emotions while maintaining empathy and ethical integrity is crucial in these moments, as it directly shapes the quality and humanity of care. How bad news is conveyed can influence not only a patient’s comprehension and decision-making processes but also their trust, dignity, and long-term psychological well-being [6].

Emotional intelligence (EI) has emerged as a key construct in this context. EI is defined as the capacity to recognise, understand, and manage emotions — both one’s own and others’. EI supports resilience, empathy, and the therapeutic relationship [7]. It also enables healthcare professionals to regulate their internal emotional states, remain present during emotionally fraught encounters, and connect with distressed patients. Studies have shown that higher emotional intelligence (EI) is associated with better teamwork, leadership, and adaptability in complex care environments [8, 9].

Despite a growing emphasis on EI and structured communication protocols such as SPIKES, there is still limited empirical research exploring how these interact in real-world clinical practice, particularly in Central and Eastern Europe. Like many countries in the region, Poland continues to modernise its healthcare education system, yet research on the emotional and communicative competencies of its healthcare workforce remains limited. In particular, it is unclear how different professional groups, such as physicians, nurses, paramedics, physiotherapists and midwives, experience and integrate these competencies into their work [10].

This study addresses this gap. Drawing on a cross-sectional sample of Polish healthcare professionals, we examined the relationships between emotional intelligence, core communication competencies, and attitudes towards breaking bad news. We aimed to assess how these constructs vary across roles, gender, experience, and institutional settings, and to understand what psychological resources underpin ethical and effective communication in challenging clinical situations.

## Methodology

### Study design

This cross-sectional observational study was conducted among healthcare professionals in Poland using an online survey. The investigation focused on communication skills, emotional intelligence, and attitudes towards breaking bad news, assessed with validated instruments: the Health Professionals Communication Skills Scale (HP-CSS), the Emotional Intelligence Questionnaire (INTE), and the Breaking Bad News Attitudes Scale (BBNAS), which measures both SPIKES adherence and openness to BBN. A priori power analysis indicated that a minimum of 384 participants was required; the final sample (*N* = 929) ensured adequate statistical power.

### Measures

#### Health professional communication skills scale (HP-CSS)

The HP-CSS is a psychometric instrument developed initially by Leal-Costa et al. (2016) to evaluate healthcare professionals’ perceived communication competencies. For this study, the authors translated and culturally adapted the tool into Polish. The Polish version comprises 18 items, which are rated on a six-point Likert scale ranging from 1 (strongly disagree) to 6 (strongly agree). The scale encompasses four dimensions: Empathy, Informative Communication, Respect and Social Skills [11].

Each subscale reflects a critical domain of professional interaction. Empathy assesses the clinician’s ability to resonate with patients emotionally; informative communication measures clarity in relaying medical information; respect evaluates non-judgmental and dignified conduct; and social skills capture the interpersonal behaviours that facilitate collaboration.

In the present sample, Cronbach’s α coefficients for HP-CSS subscales were: Empathy = 0.77, Informative Communication = 0.76, Respect = 0.73, and Social Skills = 0.72. These values are slightly lower but broadly comparable to those reported in the original validation by Leal-Costa et al. (2016), where reliability coefficients ranged from 0.72 to 0.86. The total score demonstrated acceptable internal consistency.

#### Emotional intelligence questionnaire (INTE)

Emotional intelligence was assessed using the Emotional Intelligence Questionnaire (INTE), developed and adapted for the Polish population by Jaworowska and Matczak (2005), based on the theoretical framework proposed by Salovey and Mayer (1990) [12, 13]. The instrument consists of 33 items rated on a five-point Likert scale (1 = strongly disagree to 5 = strongly agree). It provides two subscale scores: INTE I, reflecting the ability to use emotions in reasoning and behaviour, and INTE II, reflecting the ability to recognise emotions in oneself and in others. The INTE has been widely used in psychological and medical research and has demonstrated satisfactory psychometric properties [14, 15].

In healthcare, emotional intelligence is strongly linked to professional resilience, empathy, and effective communication. In this study, the INTE was used to examine differences between individuals in emotional functioning and its relationship with communication behaviour and attitudes towards delivering difficult news.

The INTE showed good reliability in our sample (α total = 0.83; INTE I = 0.78; INTE II = 0.81). These results are consistent with previous Polish validation studies (Jaworowska & Matczak, 2005), which reported α values of 0.82–0.86 for the total score [16].

#### Breaking bad news attitudes scale (BBNAS)

The BBNAS, developed by dos Santos et al. (2021), is a 15-item instrument based on the SPIKES protocol for delivering difficult medical news. It comprises two subscales: SPIKES Adherence (reflecting the respondent’s endorsement of structured communication protocols) and Openness to Breaking Bad News (capturing emotional readiness and attitudinal acceptance of this task) [17].

Items are scored on a five-point Likert scale (1 = strongly disagree to 5 = strongly agree). Higher scores indicate a more favourable attitude towards protocol use and engagement in emotionally challenging conversations. The Polish adaptation of the BBNAS has demonstrated good internal consistency (Cronbach’s alpha > 0.80) and construct validity. The Polish versions of the HP-CSS and BBNAS were developed through a forward–backward translation process and pilot-tested for clarity and internal consistency (Cronbach’s alpha > 0.80), as part of this study.

In this study, BBNAS scores were analysed about emotional intelligence (EI) and communication dimensions (HP-CSS) to identify psychosocial predictors of preparedness to deliver distressing medical information.

In the present study, the Polish version of the BBNAS showed good reliability (SPIKES adherence α = 0.78; Openness to BBN α = 0.82). These results are comparable to the original validation by dos Santos et al. (2021), where α ranged from 0.77 to 0.81.

### Data collection and procedure

Data collection was conducted between February and March 2025, following ethical approval granted on January 24, 2025 (approval no. KB-46/25, Bioethics Committee at the Regional Medical Chamber in Gdańsk). The online survey was distributed via professional websites, medical associations, institutional mailing lists, and targeted social media campaigns. Participation was voluntary, anonymous, and required electronic informed consent prior to starting the questionnaire; submission of the completed survey confirmed formal consent. To ensure data confidentiality, responses were stored on a secure server accessible only to the research team.

Eligible participants were employed healthcare professionals (physicians, nurses, paramedics, midwives, physiotherapists) with at least one year of clinical experience. Individuals not meeting these criteria were excluded. The average time to complete the survey was approximately 15 min. All procedures were conducted in accordance with the 1964 Helsinki Declaration and its later amendments or comparable ethical standards.

### Statistical analysis

Data were analysed using standard statistical techniques. Descriptive statistics were calculated for all sociodemographic and outcome variables. Comparative analyses, including one-way ANOVA and chi-square tests, were employed to examine differences between groups in communication skills, emotional intelligence and attitudes towards breaking bad news.

Pearson’s correlation coefficients were computed to explore associations between key variables. Multiple regression analyses were conducted to identify predictors of attitudes towards breaking bad news, with subscales from the HP-CSS and INTE acting as the independent variables. Regression diagnostics (e.g., residual normality, multicollinearity, homoscedasticity) were checked to ensure model validity. Statistical significance was set at *p* < 0.05. All analyses were performed using the statistical software: Statistica 13.3.

## Results

### Characteristics of participants

A total of 929 healthcare professionals completed the survey, representing a wide range of clinical roles. The largest professional groups were nurses (27.13%), paramedics (23.79%), and midwives (23.47%), followed by physicians (18.19%) and physiotherapists (7.43%). Respondents were predominantly female (74.27%) and employed mainly in clinical or university hospitals (81.16%). The mean age was 38.7 years (range 25–60). Most participants (83.1%) had between 5 and 20 years of professional experience, and 89.7% held a master’s degree or higher. Detailed demographic data are presented in Table 1.

**Table 1:** Demographic characteristics of participants

Category	Subcategory	*N* (%)
Gender	Female	690 (74.27%)
	Male	239 (25.73%)
Occupation	Nurse	252 (27.13%)
	Physician	169 (18.19%)
	Paramedic	221 (23.79%)
	Midwife	218 (23.47%)
	Physiotherapist	69 (7.43%)
Professional Experience	Less than 5 years	51 (5.49%)
	5–10 years	502 (54.04%)
	10–20 years	270 (29.06%)
	More than 20 years	106 (11.41%)
Education	Bachelor’s degree	96 (10.33%)
	Master’s degree	833 (89.67%)
Workplace	Clinical/University Hospital	754 (81.16%)
	Provincial/District Hospital	175 (18.84%)

### Descriptive statistics of key variables

Descriptive statistics for communication skills (HP-CSS), emotional intelligence (INTE), and attitudes towards breaking bad news (BBNAS) are presented in Table 2. These values (M, SD, 95% CI) provide an overview of central tendencies and variability before group comparisons, correlations, and regression analyses.

**Table 2:** Descriptive statistics (means, standard deviations, 95% CI) for communication skills (HP-CSS), emotional intelligence (INTE), and breaking bad news attitudes (BBNAS)

Variable	*N*	M	SD	−95% CI	95% CI
HP CSS					
Empathy	929	4.35	0.25	4.34	4.37
Informative Communication	929	4.81	0.32	4.79	4.84
Respect	929	4.67	0.40	4.64	4.69
Social skills	929	4.83	0.30	4.81	4.85
INTEI	929	65.42	5.00	65.09	65.74
INTEII	929	43.38	3.01	43.19	43.57
Total INTE	929	127.08	10.91	126.38	127.78
SPIKES	929	32.40	2.09	32.26	32.53
Openness to BBN	929	13.10	1.03	13.04	13.17

* INTE I – Using emotions in reasoning, INTE II – Recognising emotions, BBN – Breaking Bad News, HP-CSS – Health Professional Communication Skills Scale*

### Communication skills (HP-CSS)

Analysis of the four HP-CSS domains revealed several notable patterns.

#### Empathy

 Scores were largely consistent across professional groups. The only difference appeared by education: respondents with a bachelor’s degree reported slightly higher empathy (M = 4.40, SD = 0.25) than those with a master’s degree (M = 4.35, SD = 0.25; *p* = 0.03, d = 0.18). This was a minor effect, unlikely to carry much practical weight.

#### Informative communication

 Here the contrasts were clearer. Nurses achieved the highest scores (M = 5.06, SD = 0.30), significantly above all other groups (*p* < 0.0001, η² = 0.04). Physiotherapists also scored above physicians (*p* < 0.001, d = 0.28).

#### Respect

 Levels of respect differed slightly with experience (F = 3.45, *p* = 0.04, η² = 0.01). The youngest professionals (< 5 years) tended to score highest (M = 4.80, SD = 0.42), while those with more than two decades in practice scored lowest (M = 4.61, SD = 0.40).

#### Social skills

 These increased modestly with seniority (F = 2.89, *p* = 0.04, η² = 0.01), with early-career professionals reporting the lowest values (M = 4.73, SD = 0.30).

#### Summary

 In general, differences across groups were statistically significant but not large. Both education and professional experience appear to shape communication skills only modestly.

### Emotional intelligence (INTE)

Scores of emotional intelligence showed little variation by gender, occupation, or professional experience. The clearest difference emerged in relation to education: participants with a bachelor’s degree reported slightly higher results on INTE I (M = 66.52, SD = 5.05) compared with those holding a master’s degree (M = 65.29, SD = 4.98; *p* < 0.01, d = 0.24). A similar but weaker pattern appeared for the overall INTE score (bachelor’s: M = 129.16, SD = 11.15; master’s: M = 126.84, SD = 10.87).

Taken together, these findings indicate that emotional intelligence is fairly consistent across most groups, with education exerting only a modest influence.

Differences across professional groups were small and are detailed in Table 3.

**Table 3:** Results of one-way ANOVA for communication skills and emotional intelligence (HP-CSS and INTE) across professional groups

Variable	F	df	*p*-value
Empathy	5.12	4, 924	< 0.001
Informative Communication	4.68	4, 924	0.001
Respect	3.45	4, 924	0.008
INTE I	2.89	4, 924	0.023
INTE II	1.75	4, 924	0.135

### Attitudes towards breaking bad news (BBNAS)

Attitudes were assessed with the two BBNAS subscales: SPIKES adherence and openness to difficult conversations.

Nurses achieved the highest scores on SPIKES adherence (M = 33.44, SD = 1.59; F = 19.07, *p* < 0.0001, η² = 0.06), a moderate effect suggesting that professional role plays an important part in the uptake of structured communication strategies. In contrast, early-career professionals (0–10 years of practice) reported greater openness to breaking bad news than colleagues with 10–20 years of experience (*p* < 0.0001, d = 0.25). Although statistically significant, this effect was modest. One possible explanation is that younger clinicians, having been trained more recently, may feel more familiar with contemporary patient-centred approaches.

Education and workplace had little impact. Bachelor’s degree holders showed a tendency toward higher SPIKES adherence compared with master’s graduates (M = 32.75 vs. 32.35), but the difference did not reach significance (*p* = 0.08). No systematic variation was observed between hospital types.

Overall, nurses and younger professionals appeared somewhat more receptive to structured methods of communication, while education and workplace contributed little.

### Correlations and regression analysis

To better understand the relationships between emotional intelligence, communication skills, and attitudes toward breaking bad news, we first examined correlations and then conducted regression analyses.

Pearson’s coefficients revealed several weak but noteworthy associations. SPIKES adherence showed positive correlations with informative communication (*r* = 0.13, *p* < 0.01; 1.7% variance explained) and with INTE I – the ability to apply emotions in reasoning (*r* = 0.09, *p* < 0.05; 0.8% variance explained). Interestingly, Respect (HP-CSS) correlated negatively with openness to breaking bad news (*r* = − 0.09, *p* < 0.05; 0.8% variance explained).

While these effects reached statistical significance, they accounted for only a very small proportion of variance and should be interpreted with caution. A full overview of correlations among all variables is provided in Table 4.

**Table 4:** Pearson’s correlations between emotional intelligence, communication skills, and attitudes towards breaking bad news (*N* = 929)

Variable	INTE I	INTE II	Total INTE	Empathy	Informative Communication	Respect	SPIKES	Openness to BBN
INTE I	—	0.64**	0.91**	0.21**	0.28**	0.18*	0.30**	0.15*
INTE II	0.64**	—	0.75**	0.19*	0.25**	0.11	0.20**	0.09
Total INTE	0.91**	0.75**	—	0.22**	0.30**	0.17*	0.28**	0.13
Empathy	0.21**	0.19*	0.22**	—	0.44**	0.36**	0.18*	0.16*
Informative Communication	0.28**	0.25**	0.30**	0.44**	—	0.39**	0.35**	0.21**
Respect	0.18*	0.11	0.17*	0.36**	0.39**	—	0.22**	−0.12
SPIKES	0.30**	0.20**	0.28**	0.18*	0.35**	0.22**	—	0.27**
Openness to BBN	0.15*	0.09	0.13	0.16*	0.21**	−0.12	0.27**	—

*INTE I – Using emotions in reasoning; INTE II – Recognising emotions; BBN – Breaking Bad News; HP-CSS – Health Professional Communication Skills Scale.*

*p < 0.05, **p < 0.01, ***p < 0.001

Stepwise multiple regression models were used to identify predictors of attitudes toward breaking bad news. For SPIKES adherence, three significant predictors emerged: informative communication (β = 0.13, *p* < 0.0001), INTE I – the ability to apply emotions in reasoning (β = 0.25, *p* < 0.0001), and INTE II – recognising emotions (β = − 0.23, *p* < 0.0001). Together, these variables explained about 6% of the variance (adjusted R² = 0.06).

For general openness to breaking bad news, two predictors were identified: respect (HP-CSS) (β = − 0.09, *p* = 0.0041) and informative communication (β = 0.08, *p* = 0.0106). This model explained just 2% of the variance (adjusted R² = 0.02).

Detailed coefficients are presented in Tables 5 and 6.

**Table 5:** Stepwise regression model predicting SPIKES adherence

Predictor	β	*p*
Informative communication	0.13	< 0.0001
INTE I – using emotions in reasoning	0.25	< 0.0001
INTE II – recognising emotions	–0.23	< 0.0001

**Table 6:** Stepwise regression model predicting openness to breaking bad news

Predictor	β	*p*
Respect (HP-CSS)	–0.09	0.0041
Informative communication (HP-CSS)	0.08	0.0106

## Discussion

This study explored the interplay between communication skills, emotional intelligence, and attitudes toward breaking bad news among healthcare professionals in Poland. Using three validated instruments — the HP-CSS, INTE, and BBNAS — we examined how these domains intersect in clinical practice.

Overall, communication skills were rated highly, but some patterns emerged. Less experienced professionals reported higher levels of respect and informative communication, possibly reflecting recent curricular reforms that place greater emphasis on patient-centred approaches. Although these differences were statistically significant, their effect sizes were small, suggesting limited practical impact. However, they may still indicate broader educational trends toward empathy-oriented training, in line with earlier research showing that communication skills are shaped both by clinical exposure and the quality of training [18, 19].

Emotional intelligence also played a role. The ability to use emotions in reasoning (INTE I) predicted adherence to structured strategies, with slightly higher scores among bachelor’s graduates than master’s. One possible explanation is that undergraduate training, often more practical and interactive, may foster greater engagement with emotional aspects of clinical work than postgraduate education, which tends to be more academically oriented. Interestingly, INTE II (recognising emotions) was negatively associated with SPIKES adherence, suggesting that heightened sensitivity to patients’ feelings may sometimes discourage rigid use of protocols out of concern for rapport. This paradox aligns with research on moral distress and emotional labour in healthcare [20, 21].

The negative effect of INTE II observed in regression analyses may thus reflect a tension between empathy and standardisation, where emotionally attuned clinicians prioritise authenticity and relational understanding over protocol fidelity. Similar findings have been reported in studies linking emotional intelligence to greater resilience, stress regulation, and ethical decision-making among healthcare professionals [22].

Attitudes toward breaking bad news, measured by the BBNAS, were only modestly related to other variables. Regression models indicated that informative communication and emotional intelligence predicted adherence to SPIKES, whereas respect showed a negative association with openness to difficult conversations. This finding echoes the paradox observed in correlations, where clinicians who emphasise dignity and sensitivity appeared more hesitant to initiate distressing conversations. Such outcomes suggest that while emotional and communication competencies are important, they explain only a small proportion of readiness to deliver bad news. Broader organisational and systemic influences — such as workload, staffing, support structures, and institutional culture — are likely to exert a stronger impact.

In general, nurses and younger professionals appeared somewhat more receptive to structured methods of communication, while education and workplace contributed little to these differences. The modest nature of these effects nonetheless highlights the need for continuous communication training across all professional groups, regardless of role or seniority [23–25].

Taken together, the results highlight emotional intelligence as a clinical asset. Professionals with greater emotional awareness appear better prepared to navigate the uncertainty, vulnerability, and moral complexity of difficult conversations. Notably, gender and workplace setting had little effect, suggesting that institutional and cultural contexts may be more influential than individual demographics. The implication is clear: investing in supportive systems and training environments is likely to yield more sustainable improvements in communication practice than focusing solely on individual skills.

Finally, this study contributes novel evidence from Central and Eastern Europe, where research on communication in healthcare remains scarce. To our knowledge, it is the first study in Poland to jointly examine communication skills, emotional intelligence, and breaking bad news attitudes. It also provides preliminary psychometric support for the Polish versions of HP-CSS and BBNAS, both of which demonstrated acceptable reliability. These tools may serve as a foundation for future research and curriculum development, though further validation — including confirmatory factor analysis and test–retest reliability — remains necessary.

Ultimately, our findings reflect a broader shift in healthcare education: from purely technical proficiency toward human-centred care. Structured, reflective, and emotionally attuned training is increasingly recognised as essential. In navigating the delicate process of breaking bad news, it is not only what we say that matters, but also how we connect, reason, and respond emotionally throughout the interaction.

### Limitations

Several limitations must be considered. First, the reliance on self-report questionnaires raises concerns about social desirability and subjectivity, particularly in emotionally sensitive domains. Second, the cross-sectional design prevents causal inference: while associations and predictors were identified, it remains unclear whether improving emotional intelligence or communication skills would directly shape professional behaviour over time. Longitudinal or experimental studies are needed to clarify these relationships.

Third, although the sample was large and diverse, women constituted nearly three-quarters of participants and most respondents worked in large hospitals, which may limit generalisability to other healthcare contexts. Online recruitment may also have favoured professionals more motivated or interested in communication training. In addition, potential non-response bias cannot be excluded, as voluntary online recruitment may have led to the underrepresentation of professionals less engaged in communication training.

A further limitation concerns the psychometric tools. While the Polish versions of HP-CSS and BBNAS showed satisfactory reliability, some subscales had lower internal consistency compared with previous validation studies. Cultural differences in item interpretation or the limited number of items per subscale may partly explain this. Future research should prioritise full validation, including confirmatory factor analysis and test–retest reliability.

In addition, the present study focused on the BBNAS subscales (SPIKES adherence and Openness), as they capture theoretically distinct and practically relevant dimensions of breaking bad news. A supplementary regression model using the total BBNAS score yielded a similar pattern but with weaker explanatory power; details are available upon request. Future research should also explore how moral distress and coping strategies influence professionals’ communication behaviours in emotionally demanding contexts [21].

Finally, the use of stepwise regression carries inherent limitations, including risk of model instability and multicollinearity, which should be addressed in future research. Overall, the regression models explained only 2–6% of variance, underscoring that many important influences were not captured. Organisational, contextual, and cultural factors may be far more decisive and should be the focus of future research. Future studies should therefore integrate both individual and systemic perspectives to better capture the multidimensional nature and complexity of communication in clinical care.

## Conclusions

This study highlights the modest but meaningful role of communication skills and emotional intelligence in shaping healthcare professionals’ attitudes toward breaking bad news. While individual competencies contributed only a small share of the variance, they point to the importance of embedding structured communication and emotional training in both education and clinical practice. Equally important are system-level interventions that recognise emotional labour, provide supportive organisational cultures, and ensure conditions that allow clinicians to communicate compassionately and clearly. As the first study of its kind in Poland, these findings provide a foundation for further research and curriculum development in Central and Eastern Europe.

## Implications for practice

Our findings suggest several avenues for strengthening communication and emotional intelligence in healthcare. Training in emotional intelligence and structured approaches such as SPIKES should be integrated into both undergraduate and postgraduate education, embedded in clinical teaching rather than offered as optional modules [23–25]. Simulation-based methods, including role-play, OSCE assessments, and interprofessional high-fidelity scenarios, provide safe environments for practising challenging conversations and reflecting on mistakes without the immediate pressures of real clinical settings [23]. These abilities also require reinforcement throughout a career, for example through continuing professional development, workshops, mentoring, and reflective practice [26].

Importantly, competencies do not develop in isolation but are shaped by the culture of the workplace. Institutions that recognise the emotional labour of healthcare, provide structured debriefings after difficult events, and offer access to psychological support create conditions in which compassion and clarity can coexist. Since individual skills explained only a modest share of the variance in this study, broader system-level measures — such as adequate staffing, protected time for communication, and supportive leadership — are likely to have a more sustainable impact than training alone. Such investment not only enhances professional well-being but also improves the patient and family experience.

As the first study in Poland to jointly apply the HP-CSS, INTE, and BBNAS, these results provide an evidence-based foundation for the development of structured educational programmes in Central and Eastern Europe, where training in communication and emotional intelligence is still evolving.

## Data Availability

The datasets generated and/or analysed during the current study are available from the corresponding author on reasonable request.

## Abbreviations

BBN: Breaking bad news
EI: Emotional intelligence
HP: CSS–health professionals communication skills scale
INTE: Emotional intelligence questionnaire
BBNAS: Breaking bad news attitudes scale
SPIKES: Setting, perception, invitation, knowledge, emotions, strategy & summary (protocol)
